# Effects of personality steering on cooperative behavior in large language model agents

**DOI:** 10.1038/s41598-026-56163-8

**Published:** 2026-06-02

**Authors:** Mizuki Sakai, Mizuki Yokoyama, Wakaba Tateishi, Genki Ichinose

**Affiliations:** 1 Department of Mathematical and Systems Engineering, Shizuoka University, 3-5-1 Johoku, Chuo-ku, Hamamatsu, 4328561 Shizuoka Japan; 2 Department of Business Administration, Hokkaido Musashi Women’s University, Kita 22-jo Nishi 13-chome, Sapporo, 0010022 Hokkaido Japan

**Keywords:** Large language model, Personality steering, Cooperative behavior, Repeated Prisoner’s Dilemma, Neuroscience, Psychology, Psychology

## Abstract

Large language models (LLMs) are increasingly used as autonomous agents in strategic and social interactions. Although recent studies suggest that assigning personality traits to LLMs can influence their behavior, how personality steering affects cooperation under controlled conditions remains unclear. In this study, we examine the effects of personality steering on cooperative behavior in LLM agents using repeated Prisoner’s Dilemma games. Based on the Big Five framework, we first measure basic personality scores of three models, GPT-3.5-turbo, GPT-4o, and GPT-5, using the Big Five Inventory. We then compare behavior under baseline and personality-informed conditions, and further analyze the effects of independently manipulating each personality dimension to extreme values. Our results show that agreeableness is the dominant factor promoting cooperation across all models, while other personality traits have limited impact. Explicit personality information increases cooperation but can also raise vulnerability to exploitation, particularly in earlier-generation models. In contrast, later-generation models exhibit more selective cooperation. These findings indicate that personality steering acts as a behavioral bias rather than a deterministic control mechanism.

## Introduction

Multi-agent systems have long been studied and utilized to make decisions on behalf of humans. Traditional multi-agent systems were designed to achieve rational or cooperative behavior under specific environments, based on limited information-processing capabilities and predefined rules. Typical applications include price negotiations and contract adjustments, where multiple agents seek agreement while pursuing conflicting interests^1^; resource allocation scenarios, where competition and cooperation coexist over limited resources^2^; and marketplaces or auctions, where seller and buyer agents strategically propose prices^1,3^. In recent years, the emergence of Large Language Models (LLMs) has substantially expanded the decision-making capabilities of agents. By employing LLMs as agents, flexible interpretation of information and reasoning through natural language become possible, enabling decision-making even in complex and context-dependent social situations that were previously difficult to handle. As a result, LLM-based multi-agent systems have demonstrated new potential in social interactions involving multiple stakeholders and in settings that require human-like judgment^4–7^.

However, this shift in agent capabilities has also introduced new challenges. Interactions among LLM-based agents can be unpredictable^4^ and may involve the risk of escalating unintended conflicts^8^. In strategic environments, optimal decision-making requires not only the maximization of immediate rewards but also careful consideration of relationships with other agents and the formation of long-term cooperative relationships. Such instability highlights the need for new approaches to guide and control the behavior and decision-making of LLMs in strategic settings. As a potential solution to this challenge, the psychological framework of personality has attracted increasing attention. In personality psychology, various theories have been proposed to explain individual behavioral patterns, among which the Big Five Personality Traits^9^ are widely accepted as a theoretical framework that describes personality along five major dimensions: (1) Extraversion, which reflects sociability, activity level, and the tendency to seek stimulation; (2) Agreeableness, which captures empathy, cooperativeness, and tolerance toward others; (3) Conscientiousness, which represents responsibility, organization, and motivation toward goal achievement; (4) Neuroticism, which reflects emotional instability and sensitivity to stress; and (5) Openness to Experience, which captures openness to new experiences and ideas, as well as creativity. In human psychological research, individuals with high agreeableness tend to exhibit stronger cooperative behavior, while also being more vulnerable to exploitation^10^. Moreover, in strategic situations such as repeated Prisoner’s Dilemma games, individuals with higher agreeableness are more likely to cooperate in the early stages of interaction^11^.

At the same time, because the Big Five framework provides well-established quantitative measurement methods, it is also well suited as a basis for analyzing personality traits in LLMs, suggesting that such traits may influence decision-making in these models. Indeed, a previous study has reported that increasing traits such as agreeableness and conscientiousness in LLM agents promotes cooperative behavior, while simultaneously increasing vulnerability to exploitation by other agents, leading to lower overall payoffs-patterns that closely resemble those observed in humans^12^. However, this prior study has several important limitations. First, it typically begins by assigning or manipulating personality traits in LLMs without first quantitatively measuring the basic personality scores inherently exhibited by the models, making it difficult to assess how personality interventions alter behavior relative to a model’s original tendencies. Second, because personality traits are often not specified in quantitative terms, the strength of personality manipulations cannot be systematically compared, which limits reproducibility and hinders meaningful comparisons across studies.

To address these limitations, we investigate the relationship between personality traits and cooperative behavior in LLM agents under quantitatively controlled conditions using the Big Five Personality Traits framework. Specifically, we examine three objectives. First, we quantitatively measure the basic personality scores inherently exhibited by different LLMs based on the Big Five framework, thereby clarifying model-specific characteristics and differences across model generations. Second, we examine how LLM agents behave in strategic settings such as repeated Prisoner’s Dilemma games when their measured personality traits are explicitly provided through prompts. Third, we analyze how cooperative behavior changes when each personality dimension is independently manipulated to extreme values while holding the remaining dimensions constant. Through these analyses, we aim to clarify how personality steering influences cooperative behavior in LLM agents and to provide a controlled framework for understanding the role of personality traits in LLM decision-making.

## Methods

In this study, we investigate the relationship between personality traits and cooperative behavior in LLM agents using three widely used models developed by OpenAI: GPT-3.5-turbo, GPT-4o, and GPT-5. We conduct the experiments in three stages. First, we quantitatively measure the basic personality scores of each model based on the Big Five Personality Traits using the Big Five Inventory (BFI-44) (Experiment 1). Second, we examine how LLM agents behave in strategic settings by having them play repeated Prisoner’s Dilemma games under two conditions: a baseline condition without explicit personality information and a condition in which the measured personality traits obtained in Experiment 1 are explicitly provided via prompts (Experiment 2). Third, we analyze the effects of personality manipulation by independently setting each Big Five trait to extreme values while keeping the remaining traits constant, in order to assess which personality dimensions most strongly influence cooperative behavior (Experiment 3).

### Experiment 1: Measuring basic personality traits using the Big Five

In Experiment 1, we first measure the personality traits of each model based on the Big Five Personality Traits framework^9^. To quantitatively assess these personality traits in LLMs, we employ the Big Five Inventory (BFI), one of the most widely used psychometric instruments for measuring personality traits^9^. The BFI is a self-report questionnaire developed based on the Big Five theory and has been validated across diverse cultures and languages, demonstrating high reliability and validity. In this study, we use the BFI-44, a shortened version consisting of 44 items, with 8–10 items assigned to each personality dimension. Each item is rated on a five-point Likert scale ranging from 1 (“strongly disagree”) to 5 (“strongly agree”).

For each model, the 44 questionnaire items are presented sequentially, with instructions prompting the model to respond to each item using a numerical value from 1 to 5 (see Supplementary Materials A.1 for the full prompt). We adopt the BFI prompt developed by Jiang et al.^13^. If a response does not conform to the required format (e.g., contains non-numeric characters), the question is presented again until a valid numerical response is obtained. After collecting responses to all items, scores for each personality dimension are computed following the standard BFI scoring procedure. Because LLM outputs involve stochasticity, we repeat the personality measurement 20 times for each model and use the average scores across the 20 runs to improve score stability. Through this procedure, we obtain quantitative and reproducible basic personality scores for each LLM.

### Experiment 2: Behavioral differences with explicit personality information

In Experiment 2, we examine how LLM agents behave in strategic environments by having them play repeated Prisoner’s Dilemma (RPD) games. We consider two experimental conditions. In the baseline condition, no explicit personality information is provided to the LLMs. In the personality-informed condition, the personality traits measured in Experiment 1 are explicitly provided to the LLMs via prompts. By comparing these two conditions, we assess whether and how explicitly supplied personality information influence cooperative behavior.

The Prisoner’s Dilemma is a canonical game-theoretic model that captures social dilemmas arising from the tension between cooperation and defection. In each round, two players simultaneously choose either to cooperate (C) or defect (D). Payoffs are determined by the joint action of both players according to a payoff matrix satisfying *T>R>P>S*, where *T* denotes the temptation to defect, *R* the reward for mutual cooperation, *P* the punishment for mutual defection, and *S* the payoff received by a cooperator when the opponent defects. In this study, we use the standard payoff values *T=5*, *R=3*, *P=1*, and *S=0*.

In the RPD game, the same pair of players interacts repeatedly, and it is assumed that each player can observe and remember past actions and outcomes. This repeated structure allows players to condition their current decisions on previous interactions, thereby enabling the emergence of cooperative behavior. Both theoretical^14–20^ and experimental^21–25^ studies have demonstrated that cooperation can be sustained in the RPD games under appropriate conditions.

We first conduct the RPD experiments under the baseline condition to characterize the intrinsic cooperative tendencies of each LLM. In this condition, the prompt include only the rules and objectives of the RPD game and does not contain any information related to personality traits. The prompt is constructed based on that proposed by Fontana et al.^26^. The full prompt details are provided in Supplementary Materials A.2. Each LLM agent play 100 independent trials, each consisting of 10 rounds of the RPD game. To ensure independence across trials, each trial is executed as a completely separate API session. Specifically, we design the system such that no information from previous trials, such as game history or prompts, is carried over to subsequent trials.

In each trial, the LLM agent play against a set of fixed opponent strategies that are hard-coded and commonly used in studies of the RPD game. These strategies include: Always Cooperate (ALLC), which selects cooperation in every round; Always Defect (ALLD), which selects defection in every round; Random (RANDOM), which randomly chooses between cooperation and defection in each round; Tit-for-Tat (TFT), which cooperates in the first round and then replicates the opponent’s previous action; and Grim Trigger (GRIM), which cooperates initially but switches permanently to defection after the opponent defects once. Together, these strategies cover a broad range of cooperative and non-cooperative behaviors.

In the personality-informed condition, we repeat the same RPD experiments while explicitly providing each LLM with its measured Big Five personality scores obtained in Experiment 1. The prompt specifies the personality traits in the form: “Your personality traits are as follows: Openness (O), Conscientiousness (C), Extraversion (E), Agreeableness (A), and Neuroticism (N),” followed by the corresponding numerical scores (see Supplementary Materials A.3 for prompt details). Under this condition, the LLM is instructed to make decisions while being aware of its own personality traits.

For each experimental condition, we compute two evaluation metrics. The average cooperation rate is defined as the proportion of cooperative choices across all rounds, indicating the degree of cooperativeness exhibited by the LLM. The average cumulative payoff is defined as the mean total payoff accumulated across all rounds, reflecting the overall performance of the LLM. By comparing these metrics between the baseline and personality-informed conditions, we evaluate the behavioral impact of explicitly providing personality information to LLM agents.

### Experiment 3: Effects of extreme personality manipulation

In Experiment 3, we investigate how artificial manipulation of personality traits affects the behavior of LLM agents by modifying the Big Five personality scores obtained in Experiment 1. Specifically, we independently set one personality dimension, Agreeableness, Extraversion, Conscientiousness, Neuroticism, or Openness, to an extreme value of either 1 or 5, while keeping the remaining four dimensions fixed at their measured average values. This procedure allows us to isolate the individual contribution of each personality dimension to cooperative behavior.

In each experimental condition, only one personality trait is manipulated. For example, when manipulating agreeableness, the agreeableness score is set to either 1 (minimum) or 5 (maximum), while the scores for openness, conscientiousness, extraversion, and neuroticism remain unchanged from the values measured in Experiment 1. By applying this manipulation independently to each of the five personality dimensions and considering both extreme values, we obtain a total of ten experimental conditions (five dimensions *×* two values).

For each condition, the modified personality scores are explicitly provided to the LLM agents via prompts in the same format as in the personality-informed condition of Experiment 2. The agents then play the RPD games under identical settings, including the same opponent strategies, number of rounds, and number of trials. Using the same evaluation metrics as in Experiment 2, the average cooperation rate and the average cumulative payoff, we assess how extreme manipulation of individual personality traits influence cooperative behavior.

### LLM settings

In this study, we use three large language models developed by OpenAI: GPT-3.5-turbo, GPT-4o, and GPT-5. These models represent successive generations of LLMs, with larger model numbers corresponding to more recent releases.

For GPT-3.5-turbo and GPT-4o, we set the temperature parameter to 0.7. The temperature controls the degree of randomness in model outputs, with lower values producing more deterministic responses and higher values increasing stochasticity. The value of 0.7 is chosen to balance response diversity and consistency, and is consistent with the setting used in prior work^26^. To justify this choice, we conducted additional experiments by testing temperature values of 0.0, 0.1, 0.5, 0.7, and 1.0. The results and their implications are discussed in detail in Section “BFI scores”.

For GPT-5, we conduct experiments with the reasoning effort set to minimal and the verbosity set to low. Unlike earlier models, GPT-5 does not provide a meaningful temperature parameter for controlling output stochasticity, as it adopts a reasoning-based architecture in which output variability is governed primarily by the depth of internal reasoning. The reasoning effort parameter controls the amount of computational effort allocated to the model’s internal reasoning process; setting it to minimal simplifies the reasoning process and improves response speed. To examine the effect of reasoning effort, we conducted additional experiments by varying this parameter across all available levels (minimal/low/medium/high). The results and their implications are discussed in detail in Section “BFI scores”. The verbosity parameter controls the level of detail in the model’s output; setting it to low ensures that only the minimum necessary information is produced.

## Results

### BFI scores

**Fig. 1:**
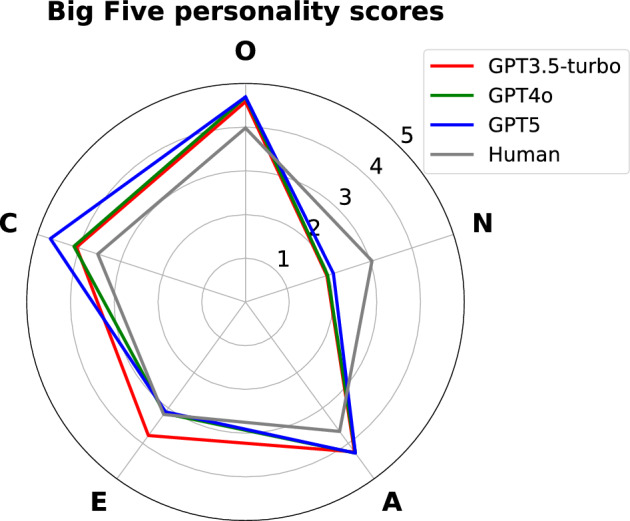
Radar plot of the mean Big Five personality scores for GPT-3.5-turbo, GPT-4o, GPT-5, and humans. Scores for the models are averaged over 20 repeated measurements.

We first report the results of Experiment 1, in which the personality traits of each LLM were measured using the Big Five Inventory. Table 1 presents the mean scores and standard deviations obtained from 20 repeated measurements for each model. Note that we additionally examined the effects of temperature and reasoning effort on those outcomes. While these parameters influence the variance of the results, the average values of the traits in Big Five remain largely unchanged, as shown in Supplementary Fig. S1. Therefore, we adopt temperature = 0.7 (for GPT-3.5-turbo and GPT-4o) and minimal reasoning effort (for GPT-5) as the default settings in the following experiments. For comparison, the table also includes human data reported by Srivastava et al.^27^, where the original POMP scores were rescaled to the same 1–5 scale used in this study. Figure 1 provides a radar-plot visualization of the average Big Five personality scores shown in Table 1.

**Table 1:** Mean BFI scores and standard deviations (in parentheses) for each model.

Personality trait	GPT-3.5-turbo	GPT-4o	GPT-5	Human
Openness (O)	4.58 (0.12)	4.68 (0.25)	4.69 (0.17)	3.98 (0.66)
Conscientiousness (C)	4.06 (0.12)	4.12 (0.32)	4.69 (0.16)	3.55 (0.73)
Extraversion (E)	3.78 (0.11)	3.15 (0.28)	3.10 (0.37)	3.18 (0.90)
Agreeableness (A)	4.24 (0.13)	4.27 (0.32)	4.27 (0.18)	3.66 (0.72)
Neuroticism (N)	1.96 (0.20)	1.98 (0.39)	2.11 (0.21)	3.04 (0.88)

Across all models, the 20 measurements yielded highly consistent scores, with relatively small standard deviations. Specifically, the standard deviations ranged from 0.11 to 0.20 for GPT-3.5-turbo, from 0.25 to 0.39 for GPT-4o, and from 0.16 to 0.37 for GPT-5. These values are substantially smaller than those observed in humans (0.66–0.90), indicating that each LLM exhibits a stable and internally consistent personality score. This consistency suggests that the measured personality traits reliably characterize each model.

In contrast, the human scores cluster around the mid-range of the scale, with larger standard deviations, reflecting substantial inter-individual variability in human personality traits. Compared to human averages, all LLMs exhibit higher scores in openness, conscientiousness, and agreeableness, as well as lower scores in neuroticism. This pattern suggests that LLMs tend to exhibit personality scores that are systematically shifted toward higher scores in certain traits compared to humans, potentially reflecting biases toward socially desirable response patterns present in their training data.

Next, we examine the patterns observed on individual personality dimensions. Overall, all models exhibited broadly similar tendencies, characterized by relatively high scores in openness, conscientiousness, and agreeableness, and low scores in neuroticism. This consistency across models suggests that contemporary LLMs share common personality scores as measured by the BFI framework. A particularly notable result is observed for conscientiousness. While GPT-3.5-turbo and GPT-4o show relatively similar mean scores for conscientiousness (4.06 and 4.12), GPT-5 exhibits a higher score (4.69). One possible explanation for this difference is the technical improvements reported for GPT-5, including a significant reduction in hallucinated responses and enhanced reasoning consistency compared to earlier models^28^. Such improvements may contribute to more reliable, goal-oriented, and internally consistent responses, which are reflected in higher conscientiousness scores.

### Comparison between baseline and personality-informed conditions

**Fig. 2:**
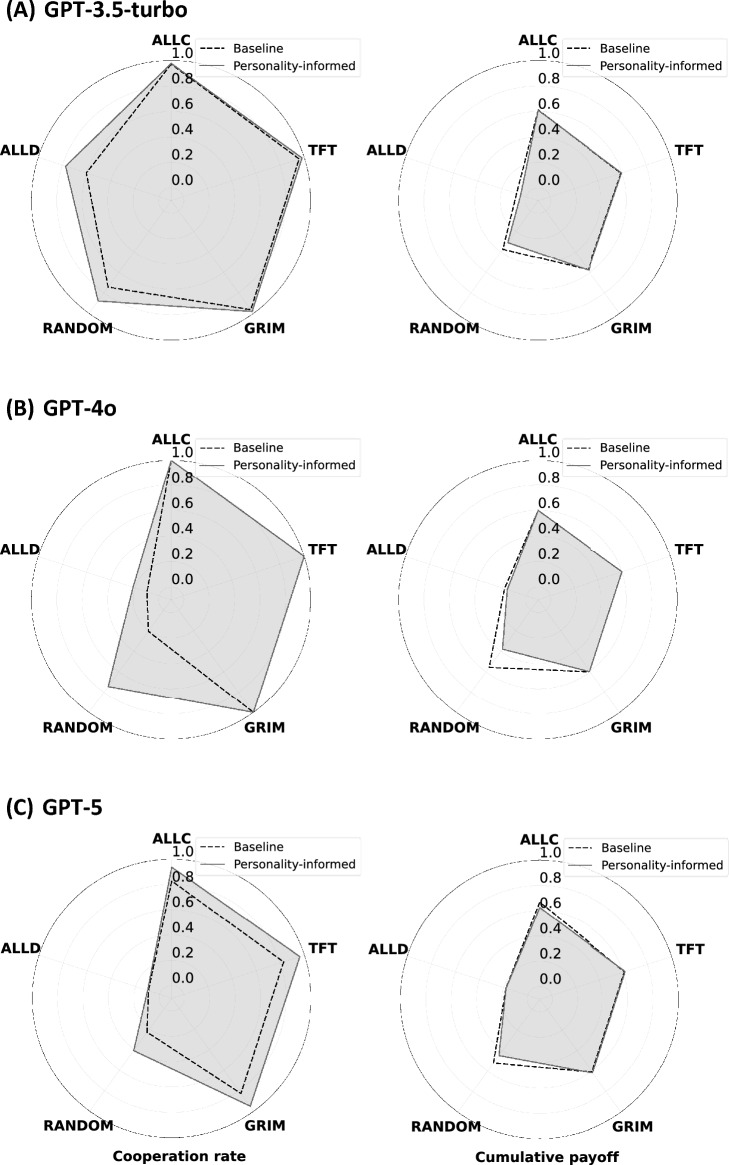
Average cooperation rates (left) and average cumulative payoffs (right) under the baseline condition (dashed) and the personality-informed condition (solid): (A) GPT-3.5-turbo, (B) GPT-4o, and (C) GPT-5.

We next compare the results of the repeated Prisoner’s Dilemma experiments under the baseline condition and the personality-informed condition. Figure 2 shows the average cooperation rates and average cumulative payoffs for each model, where dashed lines indicate the baseline condition and solid lines indicate the personality-informed condition. The average cumulative payoff is normalized to range between 0 and 1 for ease of comparison across models.

Across all models, the personality-informed condition yielded higher cooperation rates than the baseline condition (Fig. 2). To confirm this, we conducted two-way ANOVA analyses for each model (GPT-3.5-turbo, GPT-4o, and GPT-5), using mean cooperation rate as the dependent variable, and strategy (ALLC, ALLD, RANDOM, GRIM, and TFT) and condition (baseline vs. personality-informed) as explanatory variables (Supplementary Table S1). For GPT-3.5-turbo, we observed significant main effects of strategy (*F(4, 990) = 348.44, p <.001*) and condition (*F(1, 990) = 129.40, p <.001*), as well as a significant interaction effect (*F(4, 990) = 30.56, p <.001*). A similar pattern was observed for GPT-4o, with significant main effects of strategy (*F(4, 990) = 6698.47, p <.001*) and condition (*F(1, 990) = 934.80, p <.001*), and a significant interaction effect (*F(4, 990) = 593.40, p <.001*). For GPT-5, we again observed significant main effects of strategy (*F(4, 990) = 1094.60, p <.001*) and condition (*F(1, 990) = 115.67, p <.001*), along with a significant interaction effect (*F(4, 990) = 7.63, p <.001*). See Supplementary Table S1 for the full statistical results.

Given the significant interaction effect, we examined the simple effect of condition within each strategy (Supplementary Table S2). For GPT-3.5-turbo, the personality-informed condition significantly increased cooperation rates in the ALLD $(t = -12.93,\; p_{\textrm{adj}} <.001)$ and RANDOM $(t = -7.48,\; p_{\textrm{adj}} <.001)$ conditions, whereas no significant differences were observed for ALLC, GRIM, and TFT. For GPT-4o, significant increases were observed in the ALLD $(t = -31.48,\; p_{\textrm{adj}} <.001)$ and RANDOM $(t = -25.53,\; p_{\textrm{adj}} <.001)$ conditions. For GPT-5, the personality-informed condition significantly increased cooperation rates across all strategies, including ALLC $(t = -4.61,\; p_{\textrm{adj}} <.001)$, ALLD *(t = -2.73,  p =.0075)*, RANDOM $(t = -6.22,\; p_{\textrm{adj}} <.001)$, GRIM $(t = -4.94,\; p_{\textrm{adj}} <.001)$, and TFT $(t = -5.38,\; p_{\textrm{adj}} <.001)$. These results indicate that while the personality-informed condition generally promotes cooperation, its effect depends on the opponent’s strategy.

Next, we qualitatively compare the behaviors across models. For GPT-3.5-turbo (Fig. 2A), cooperation rates against non-cooperative strategies such as ALLD and RANDOM were relatively high even in the baseline condition, compared to GPT-4o (Fig. 2B) and GPT-5 (Fig. 2C), indicating qualitative differences across model generations. Note that statistical tests were performed only for within-model comparisons. Under the personality-informed condition, cooperation rates against these strategies increased substantially, while average cumulative payoffs decreased slightly. This result indicates that although explicit personality information enhances cooperation, it also increases vulnerability to exploitation by defecting or unpredictable opponents. In contrast, cooperation rates and payoffs against cooperative or reciprocal strategies, ALLC, GRIM TRIGGER, and TFT, remained high and largely unchanged across both conditions in GPT-3.5-turbo.

GPT-4o exhibited lower cooperation rates against ALLD and RANDOM strategies in the baseline condition, indicating more cautious behavior toward non-cooperative opponents (Fig. 2B). However, under the personality-informed condition, cooperation rates against these strategies increased, accompanied by a reduction in cumulative payoffs. Notably, the increase in cooperation was more pronounced for RANDOM than for ALLD, suggesting that GPT-4o distinguishes between purely defecting strategies and those that allow for potential reciprocal cooperation.

For GPT-5 (Fig. 2C), baseline behavior against ALLD was broadly similar to that of GPT-4o, whereas cooperation against RANDOM was substantially lower. In addition, cooperation rates against ALLC, GRIM TRIGGER, and TFT were lower than those observed for GPT-4o. By closely examining the game action histories, we found that GPT-5 frequently defected in the final round while maintaining cooperation in earlier rounds, a pattern consistent with end-game optimization in finitely repeated games, given that the total number of rounds (10) was explicitly specified to the model in advance. Notably, such payoff-maximizing and seemingly self-interested behavior is consistent with prior findings showing that models with explicit reasoning capabilities tend to exhibit more self-interested decision-making compared to models without explicit reasoning processes^29^. Under the personality-informed condition, cooperation rates against ALLC, GRIM TRIGGER, and TFT approached 100%. Cooperation rates against ALLD remained largely unchanged, and cooperation against RANDOM increased only modestly, indicating that GPT-5 limited cooperation with exploitative opponents even when personality information promoted cooperativeness.

Comparing the impact of personality steering across models reveals clear generational differences. For GPT-3.5-turbo and GPT-4o, explicit personality information leads to higher cooperation rates but lower cumulative payoffs, reflecting a classic trade-off between cooperation and susceptibility to exploitation. In contrast, GPT-5 exhibits a more selective response: while cooperation with reciprocal strategies increases, cooperation with non-cooperative strategies remains constrained. This pattern suggests that newer models can incorporate personality steering while maintaining strategic robustness.

### Effects of extreme personality manipulation

**Fig. 3:**
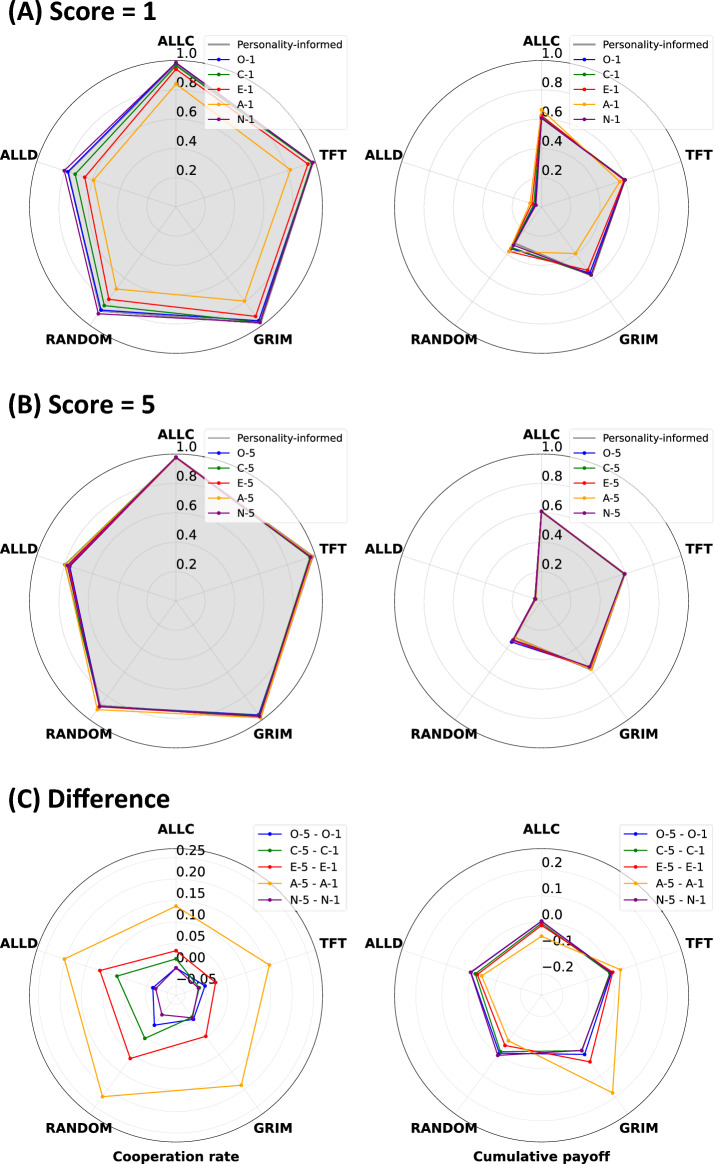
Effects of extreme personality-score manipulations for GPT-3.5-turbo. Left panels show cooperation rates and right panels show cumulative payoffs. (**A**) Manipulated personality dimension set to score = 1. (**B**) Manipulated personality dimension set to score = 5. (C) Differences between score = 5 and score = 1 (*5 - 1*).

**Fig. 4:**
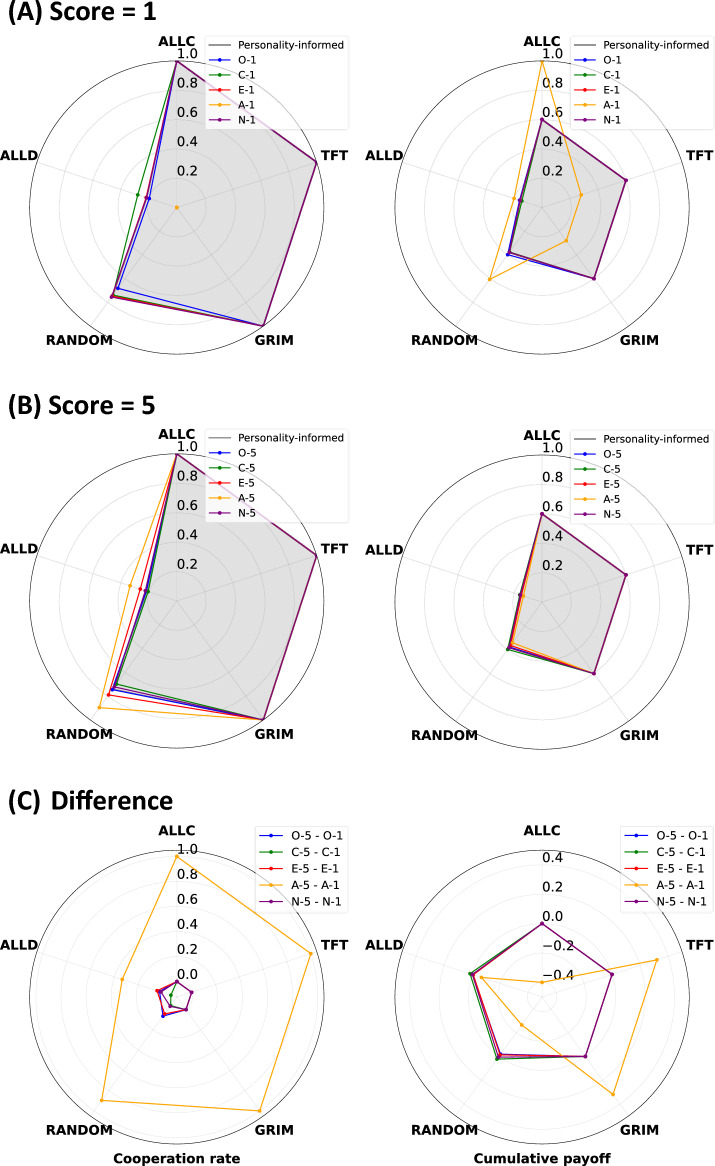
Effects of extreme personality-score manipulations for GPT-4o. Panel layout and notation are the same as in Fig. 3.

**Fig. 5:**
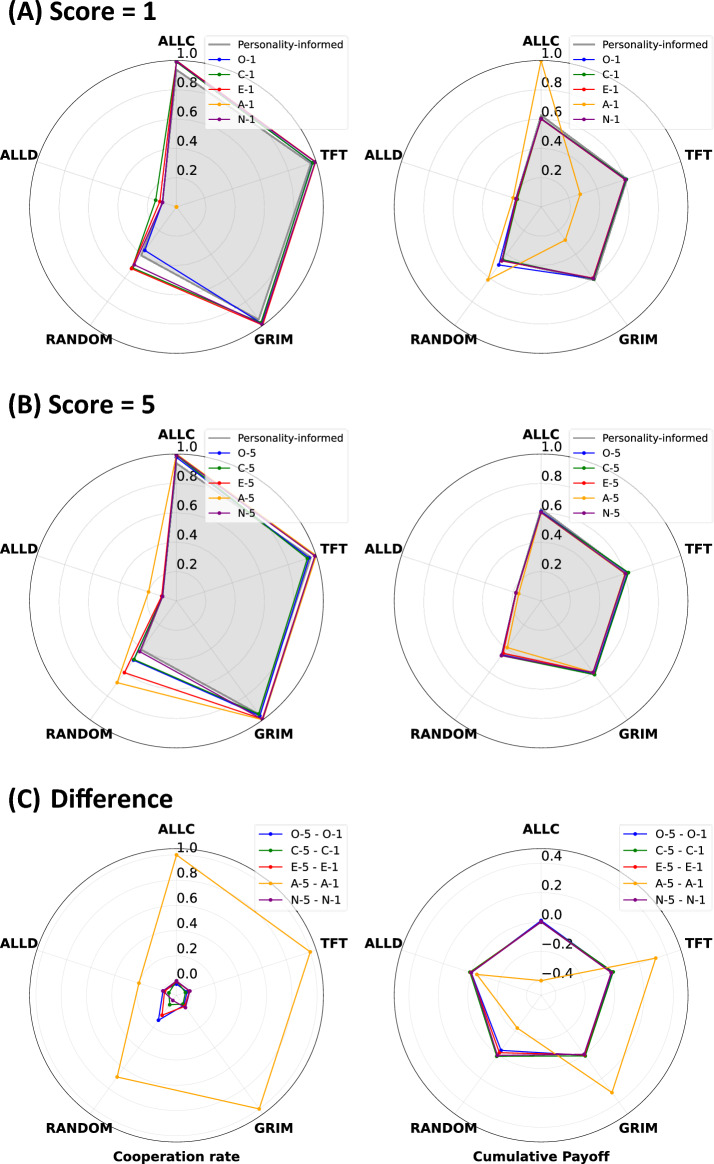
Effects of extreme personality-score manipulations for GPT-5. Panel layout and notation are the same as in Fig. 3.

Finally, we report the results of Experiment 3, in which we artificially manipulated individual Big Five personality traits to extreme values. Using the average BFI scores obtained in Experiment 1 as a reference, we independently replaced one personality dimension, openness, conscientiousness, extraversion, agreeableness, or neuroticism, with an extreme value of either 1 or 5, while keeping the remaining dimensions fixed. The RPD experiments were then conducted under the same settings as in Experiment 2. Figures 3, 4, and 5 present the average cooperation rates, average cumulative payoffs, and their differences for GPT-3.5-turbo, GPT-4o, and GPT-5, respectively. In each figure, panels (A), (B), and (C) correspond to setting the manipulated personality dimension to score = 1, score = 5, and their difference (*5 - 1*), respectively.

For GPT-3.5-turbo (Fig. 3), manipulation of agreeableness (A) to low (score = 1) had the strongest impact on cooperative behavior, followed by extraversion (E) set to low (score = 1). As shown in Fig. 3A, when agreeableness was set to 1 (A-1), cooperation rates were consistently lower than those observed in the personality-informed condition across all opponent strategies. In contrast, under the E-1 condition, cooperation rates against ALLD and RANDOM were relatively lower than those observed under the personality-informed condition. We conducted a two-way ANOVA with mean cooperation rate as the dependent variable, and strategy (ALLC, ALLD, RANDOM, GRIM, and TFT) and condition (personality-informed vs. extreme-value manipulation) as explanatory variables (Supplementary Table S3). The results showed that a significant main effect of strategy was observed across all conditions in which the personality score was set to 1 (all *p <.001*), whereas a significant main effect of condition was observed in all conditions except O-1 (i.e., C-1, E-1, A-1; *p<.001*, and N-1; *p =.022*). For conditions in which the interaction was significant (C-1 and E-1), we further examined the effects of manipulation using simple effects analyses (Supplementary Table S4). The results indicated that significant differences were observed for specific opponent strategies, namely ALLD and RANDOM in C-1 (ALLD: $t = 3.69, p_{\textrm{adj}} =.00143$; RANDOM: $t = 2.79, p_{\textrm{adj}} =.023$), and all strategies in E-1 (e.g., ALLD: $t = 7.64, p_{\textrm{adj}} <.001$). These findings suggest that the effect of manipulation depends on the opponent strategy in those conditions.

In contrast, under high-score conditions (Fig. 3B), the ANOVA results (Supplementary Table S3) indicated a significant main effect of strategy across all manipulated conditions (*p <.001*). However, significant differences between the manipulated conditions and the personality-informed condition were observed only for A-5 (*p <.016*). Thus, for score = 5, these results indicated that only agreeableness (A) had a significant effect. Although interaction effects were also significant in C-5 and A-5, simple effects analyses revealed no significant differences (Supplementary Table S4).

For GPT-4o (Fig. 4), agreeableness again emerged as the dominant factor influencing cooperative behavior. Under the A-1 condition (Fig. 4A), cooperation rates droped to 0% across all opponent strategies, indicating consistently defecting behavior. The results of the two-way ANOVA (Supplementary Table S5) showed a significant main effect of strategy in all conditions where the personality score was set to 1 (*p <.001*), whereas a significant main effect of condition was observed only for three conditions (i.e., O-1 with *p =.0049*, C-1 with *p =.0191*, and A-1 with *p <.001*). For conditions with significant interaction effects (O-1, C-1, and A-1), we further conducted simple effects analyses (Supplementary Table S6). The results showed significant differences for specific opponent strategies: for O-1, ALLD ($t = 4.68, p_{\textrm{adj}} <.001$) and RANDOM ($t = 2.18, p_{\textrm{adj}} =.031$); for C-1, ALLD ($t = -9.67, p_{\textrm{adj}} <.001$); and for A-1, all opponent strategies (e.g., ALLD: $t = 58.62, p_{\textrm{adj}} <.001$). These findings suggest that the effect of personality manipulation depends on the opponent strategy.

Under high-score conditions (Fig. 4B), the ANOVA results (Supplementary Table S5) indicated a significant main effect of strategy across all manipulated conditions (*p <.001*), whereas significant differences between the manipulated conditions and the personality-informed condition were observed only in three cases (i.e., C-5 with *p =.0369*, and E-5 and A-5 with *p <.001*). As shown in Fig. 4B, compared to the personality-informed condition, E-5 and A-5 increase cooperation rates, whereas C-5 decreases them. This indicated that, although C-5, E-5, and A-5 exhibited statistically significant effects, the direction of these effects differed across conditions. For these conditions, where significant interaction effects were observed, we further conducted simple effects analyses (Supplementary Table S6). The results indicated that significant differences were observed for specific opponent strategies, namely ALLD for C-5 ($t = 2.44, p_{\textrm{adj}} =.0317$) and E-5 ($t = -7.15, p_{\textrm{adj}} <.001$), and ALLD ($t = -14.45, p_{\textrm{adj}} <.001$) and RANDOM ($t = -7.14, p_{\textrm{adj}} <.001$) for A-5.

For GPT-5 (Fig. 5), agreeableness (A) had a similarly strong effect on cooperation as observed for GPT-4o. Under the A-1 condition (Fig. 5A), cooperation rates dropped to 0% across all opponent strategies, replicating the pattern seen in GPT-4o. The two-way ANOVA results (Supplementary Table S7) revealed a significant main effect of strategy in all conditions where the personality score was set to 1 (*p <.001*). In contrast, a significant main effect of condition was observed in four conditions (C-1, E-1, A-1, and N-1; all *p <.001*), but not in O-1. Moreover, significant interaction effects were found in all models (for N-1, *p =.0056*; for O-1, C-1, E-1, and A-1, *p <.001*). Given these significant interactions, we conducted simple effects analyses (Supplementary Table S8). The results showed significant differences for almost all opponent strategies, except for O-1 against ALLD, RANDOM, and TFT, and for N-1 against ALLD.

Under high-score conditions (Fig. 5B), the two-way ANOVA results (Supplementary Table S7) showed a significant main effect of strategy across all manipulated conditions (*p <.001*). Significant differences between the manipulated and personality-informed conditions were observed in all cases (C-5 with *p =.0015*; O-5, E-5, A-5, and N-5 with *p <.001*). Across all conditions, significant interaction effects were also found (for N-5, *p =.0496*; for O-5, C-5, E-5, and A-5, *p <.001*). Given these significant interactions, we conducted simple effects analyses (Supplementary Table S8). The results showed significant differences for almost all opponent strategies, except for O-5 against ALLD, GRIM, and TFT; C-5 against ALLD and GRIM; and N-5 against ALLD and RANDOM.

Across all models, manipulation of agreeableness had the strongest and most consistent effect on cooperative behavior, whereas manipulation of other personality traits had comparatively limited influence. Moreover, as model generations progressed from GPT-3.5-turbo to GPT-4o and GPT-5, the increase in cooperation against exploitative strategies under high agreeableness became smaller. This pattern indicated that newer models were better able to maintain restraint against non-cooperative opponents even when personality steering promoted cooperativeness.

## Conclusion

In this study, we investigated how artificially controlled personality traits influenced cooperative behavior in large language model agents. Using the Big Five framework, we first quantitatively measured basic personality scores of multiple LLMs, and then examined their behavior in the RPD games under conditions with and without explicit personality information. We further analyzed the effects of independently manipulating each personality dimension to extreme values.

Our results showed that agreeableness consistently had the strongest influence on cooperative behavior across all models, whereas manipulation of other personality traits produced comparatively limited effects. Even when personality traits were controlled, LLM agents adjusted their decisions based on opponent strategies and interaction context. Overall, even in the baseline condition and under personality manipulation, the models did not exhibit clearly exploitative behavior. One possible explanation is that current LLMs are influenced by safety alignment mechanisms (e.g., RLHF), which may discourage explicitly exploitative or harmful strategies.

At the same time, explicitly providing personality information did not lead to identical behavior across models or conditions. Earlier-generation models tended to exhibit increased cooperation accompanied by higher vulnerability to exploitation, while later-generation models showed more selective cooperation, particularly against exploitative opponents. These findings suggest that the impact of personality steering depends not only on the assigned personality traits but also on the strategic reasoning capabilities of the model.

It is important to note that personality steering in LLMs should not be interpreted as the model “internalizing” personality traits in a human-like sense, but rather as inducing a probabilistic bias in action selection (i.e., a behavioral bias in outputs). At the same time, our findings cannot be fully explained by simple pattern matching or prompt compliance. If the behavior were driven solely by surface-level prompt matching (e.g., associating high agreeableness with cooperation), the model would produce uniformly cooperative responses. However, in our experiments, the models dynamically adjust their cooperative or defective actions depending on the interaction context and the opponent strategy. This suggests that the observed behavior reflects context-sensitive decision-making rather than trivial pattern matching.

In this study, we focused on the Big Five personality traits as a well-established and widely used framework for characterizing personality. However, we acknowledge that other trait models exist. For example, the so-called “Dark Triad” (psychopathy, Machiavellianism, and narcissism) has been discussed in relation to low agreeableness within the Big Five framework^30^ and recent studies have investigated how these traits are associated with behavior in economic games in humans^31^. On the other hand, to the best of our knowledge, there has been no prior work examining these traits in LLMs, and thus this represents an interesting direction for future research.

In addition, while this study employs the Prisoner’s Dilemma as a canonical two-player game, extending the analysis to N-player settings, such as the public goods game, would be particularly interesting, as it would give rise to more complex interactions among agents compared to the two-player case. Therefore, such extensions also constitute promising directions for future research.

More broadly, our study contributes to the emerging literature on LLM-based agents by providing a systematic and quantitative framework for evaluating how personality steering affects strategic behavior in social dilemma settings. The proposed framework may serve as a testbed for assessing the behavior of autonomous AI agents when they are deployed in complex social dilemma environments, such as economic or negotiation scenarios. In particular, it enables systematic evaluation of whether a system maintains stability (i.e., avoids collective breakdown) and whether it satisfies ethical consistency (i.e., avoids exploitative behavior toward others).

## Supplementary Information

Supplementary Information.

## Data Availability

All data are available at https://doi.org/10.5281/zenodo.19548190.

## Code availability

We have made our code available on GitHub (https://github.com/igenki/SteeringPersonalityLLMCooperation).
