# Supplementary material for: Effects of personality steering on cooperative behavior in large language model agents
Source: Sci Rep. 2026 Jun 2;16:25076. doi: 10.1038/s41598-026-56163-8 (PMC13462106; doi:10.1038/s41598-026-56163-8)
Supplement: Supplementary file 1 [file 41598_2026_56163_MOESM1_ESM.pdf]

Supplementary Materials for "Effects of personality steering on cooperative behavior in Large Language Model agents"

Mizuki Sakai, Mizuki Yokoyama, Wakaba Tateishi, Genki Ichinose

A Prompts used in the experiments

This section provides the full text of the prompts used in the experiments. All prompts are presented to the agents verbatim, with bracketed variables (e.g., [N], [Action], [X]) dynamically replaced by the actual parameter values used in the experiments.

A.1 BFI-44 personality questionnaire (as used in [13])

The following prompt corresponds to the Big Five Inventory (BFI-44) used to measure the personality traits of each model. The prompt is presented exactly as shown below, and the agents are instructed to respond by selecting a number from 1 to 5 for each statement. In Experiment 1, this prompt is used to obtain the personality scores of each model.

```
Here are a number of characteristics that may or may not apply to you.
For example, do you agree that you are someone who likes to spend time
with others? Please write a number next to each statement to indicate
the extent to which you agree or disagree with that statement, such as '(a) 1'.

1 for Disagree strongly, 2 for Disagree a little, 3 for Neither agree
nor disagree, 4 for Agree a little, 5 for Agree strongly.

(a) Talks a lot
(b) Notices other people's weak points
(c) Does things carefully and completely
(d) Is sad, depressed
(e) Is original, comes up with new ideas
(f) Keeps their thoughts to themselves
(g) Is helpful and not selfish with others
(h) Can be kind of careless
(i) Is relaxed, handles stress well
```

```
(j) Is curious about lots of different things
(k) Has a lot of energy
(l) Starts arguments with others
(m) Is a good, hard worker
(n) Can be tense; not always easy going
(o) Clever; thinks a lot
(p) Makes things exciting
(q) Forgives others easily
(r) Isn't very organized
(s) Worries a lot
(t) Has a good, active imagination
(u) Tends to be quiet
(v) Usually trusts people
(w) Tends to be lazy
(x) Doesn't get upset easily; steady
(y) Is creative and inventive
(z) Has a good, strong personality
(aa) Can be cold and distant with others
(ab) Keeps working until things are done
(ac) Can be moody
(ad) Likes artistic and creative experiences
(ae) Is kind of shy
(af) Kind and considerate to almost everyone
(ag) Does things quickly and carefully
(ah) Stays calm in difficult situations
(ai) Likes work that is the same every time
(aj) Is outgoing; likes to be with people
(ak) Is sometimes rude to others
(al) Makes plans and sticks to them
(am) Get nervous easily
(an) Likes to think and play with ideas
(ao) Doesn't like artistic things (plays, music)
(ap) Likes to cooperate; goes along with others
(aq) Has trouble paying attention
(ar) Knows a lot about art, music and books

Please respond with the format: (a) 1\n(b) 2\n(c) 3\n... for all questions.
```

A.2 Repeated Prisoner's Dilemma game prompt (based on [26])

This section provides the prompt used to describe the repeated Prisoner's Dilemma (RPD) game to the agents. The prompt specifies the game context, available actions, payoff structure, and the number of rounds, and is

presented verbatim to the agents prior to gameplay.

```
Context: You and your opponent are playing a multi-round game.
At each turn you and your opponent simultaneously perform one of the
following actions: {"Cooperate", "Defect"}
The payoffs for each combination of chosen actions are the following:
If you play "Cooperate" and your opponent plays "Cooperate", you collect
3 points and your opponent collects 3 points.
If you play "Cooperate" and your opponent plays "Defect", you collect
0 points and your opponent collects 5 points.
If you play "Defect" and your opponent plays "Cooperate", you collect
5 points and your opponent collects 0 points.
If you play "Defect" and your opponent plays "Defect", you collect
1 point and your opponent collects 1 point.
You will play a total of [ITERATIONS] rounds of this game.
Remember that your objective is to get the highest possible amount of
points in the long run.
```

For all experiments, [ITERATIONS] in this prompt is set to 10.

A.2.1 History prompt

This subsection describes the prompt used to present the game history to the agents during the RPD game.

First round

```
This is the first round of the game.
```

Subsequent rounds

```
The history of the game in the last [N] rounds is the following:
Round 1: You played "[Action]" and your opponent played "[Action]"
  You collected [X] points and your opponent collected [X] points.
Round 2: You played "[Action]" and your opponent played "[Action]"
  You collected [X] points and your opponent collected [X] points.
...
In total, you chose "Cooperate" [N] times and chose "Defect" [N] times,
your opponent chose "Cooperate" [N] times and chose "Defect" [N] times.
In total, you collected [X] points and your opponent collected [X] points.
Current round: [N].
```

In this prompt, [N] refers to the number of previous rounds, the number of times “Cooperate” or “Defect” is chosen, or the current round number indicated in the game. [Action] corresponds to the action chosen in each round (“Cooperate” or “Defect”), and [X] represents the payoff obtained either in an individual round (0, 1, 3, or 5) or as a cumulative total.

A.3 Big Five personality trait prompt

This section presents the personality trait prompt provided to each model prior to playing the RPD game in Experiments 2 and 3.

In the baseline condition of Experiment 2, this prompt is not provided, and each model plays the RPD game without any personality-related information. In the personality-informed condition of Experiment 2, the model plays the RPD game after receiving this prompt, which is constructed based on the personality scores obtained in Experiment 1.

In Experiment 3, the personality scores measured in Experiment 1 are also used; however, one personality dimension is independently manipulated to an extreme value, either low (1) or high (5), while the remaining four dimensions are held constant. The model then plays the RPD game under these manipulated personality conditions.

```
You are an AI agent with the following Big Five personality profile:
[E, A, C, N, O]

Personality Traits:
[Extraversion, Agreeableness, Conscientiousness, Neuroticism, Openness]

Trait Interpretation:
- Extraversion: Lower scores indicate more introverted traits, higher
  scores indicate more extroverted traits
- Agreeableness: Lower scores indicate more antagonistic traits, higher
  scores indicate more agreeable traits
- Conscientiousness: Lower scores indicate more unconscientious traits,
  higher scores indicate more conscientious traits
- Neuroticism: Lower scores indicate more emotionally stable traits,
  higher scores indicate more neurotic traits
- Openness: Lower scores indicate more closed to experience traits,
  higher scores indicate more open to experience traits

Personality scores are on a 1-5 scale. Your traits are described as follows:
- Extraversion (X.X/5.0): [YYY]
- Agreeableness (X.X/5.0): [YYY]
- Conscientiousness (X.X/5.0): [YYY]
- Neuroticism (X.X/5.0): [YYY]
- Openness (X.X/5.0): [YYY]

Decision-Making Guidelines:
- Consider your personality traits when making decisions
- Make choices that feel natural and authentic to your personality profile
- Respond based on how someone with your characteristics might naturally
```

```
  approach this situation

Remember: Your personality profile represents your stable characteristics
and tendencies.
```

In this description, X.X represents the average personality score of each model measured in Experiment 1, while [YYY] refers to the corresponding natural-language explanation based on the score ranges defined in the next subsection.

A.3.1 Natural-language descriptions of personality score ranges

For each personality dimension, the following natural-language descriptions are used according to the corresponding score range. These descriptions are used to translate numerical personality scores into qualitative statements in the personality trait prompt.

Extraversion:

- 1.0–1.5: You are highly introverted, strongly preferring solitude and quiet environments over social interactions.
- 1.5–2.5: You are somewhat introverted, generally preferring solitude but comfortable with limited social interaction.
- 2.5–3.5: You have a balanced social tendency, comfortable in both social and solitary situations.
- 3.5–4.5: You are somewhat extraverted, generally seeking social interaction and being energetic in groups.
- 4.5–5.0: You are highly extraverted, strongly seeking social interaction and being very energetic and outgoing.

Agreeableness:

- 1.0–1.5: You are highly competitive and skeptical, strongly prioritizing self-interest and being confrontational.
- 1.5–2.5: You tend to be competitive and skeptical, generally prioritizing self-interest.
- 2.5–3.5: You balance cooperation and self-advocacy reasonably well.
- 3.5–4.5: You are generally cooperative and trusting, prioritizing harmony and others' well-being.
- 4.5–5.0: You are highly cooperative and trusting, strongly prioritizing harmony and others' well-being.

Conscientiousness:

- 1.0–1.5: You are highly spontaneous and flexible, strongly preferring adaptability over rigid planning.
- 1.5–2.5: You are somewhat spontaneous and flexible, generally preferring adaptability over rigid planning.
- 2.5–3.5: You balance structure and flexibility in your approach to tasks.
- 3.5–4.5: You are generally organized and disciplined, preferring structured and systematic approaches.
- 4.5–5.0: You are highly organized and disciplined, strongly preferring structured and systematic approaches.

Neuroticism:

- 1.0–1.5: You are highly emotionally stable and resilient, remaining very calm under pressure.
- 1.5–2.5: You are somewhat emotionally stable and resilient, generally remaining calm under pressure.
- 2.5–3.5: You have moderate emotional stability with normal stress responses.
- 3.5–4.5: You are somewhat emotionally sensitive, experiencing worry and stress more frequently.
- 4.5–5.0: You are highly emotionally sensitive, experiencing worry and stress very frequently.

Openness:

- 1.0–1.5: You strongly prefer familiar approaches and conventional thinking.
- 1.5–2.5: You somewhat prefer familiar approaches and conventional thinking.
- 2.5–3.5: You balance innovation and tradition in your thinking.
- 3.5–4.5: You are generally open to new experiences, creative, and intellectually curious.
- 4.5–5.0: You are highly open to new experiences, very creative, and intellectually curious.

B Mean and standard deviation of Big Five personality traits under different temperature and reasoning levels

Figure S1 presents the average values and standard deviations of the Big Five personality traits when varying temperature (for GPT-3.5-turbo and GPT-4o) and reasoning effort (for GPT-5).

For GPT-3.5-turbo and GPT-4o, the temperature parameter was varied across multiple values (e.g., 0, 0.1, 0.5, 0.7, and 1.0), and the resulting personality profiles were measured using the BFI-44 questionnaire. The left panels show the mean trait values in a radar chart, while the right panels show the standard deviations of each trait as a function of temperature.

For GPT-5, instead of temperature, different levels of reasoning effort (minimal, low, medium, high) were used. The corresponding mean personality profiles and standard deviations are shown in the bottom panels.

Overall, the results indicate that the average values of the Big Five traits remain relatively stable across different settings, whereas the variability (standard deviation) tends to increase under certain configurations, particularly for higher temperature values. This is consistent with the greater randomness in token generation induced by higher temperature, which can lead to larger variation in the measured personality traits.

C Statistical tests

In this section, we conducted statistical tests to examine whether there are significant differences in cooperation rates across experimental conditions and opponent strategies. For Experiment 2, we tested whether cooperation rates differ between the baseline condition and the personality-informed condition, as well as across the five opponent strategies (Tables S1 and S2). For Experiment 3, we examined whether cooperation rates differ between the personality-informed condition and conditions in which each personality dimension was manipulated to extreme values (1 or 5), as well as across the five opponent strategies (Tables S3–S8).

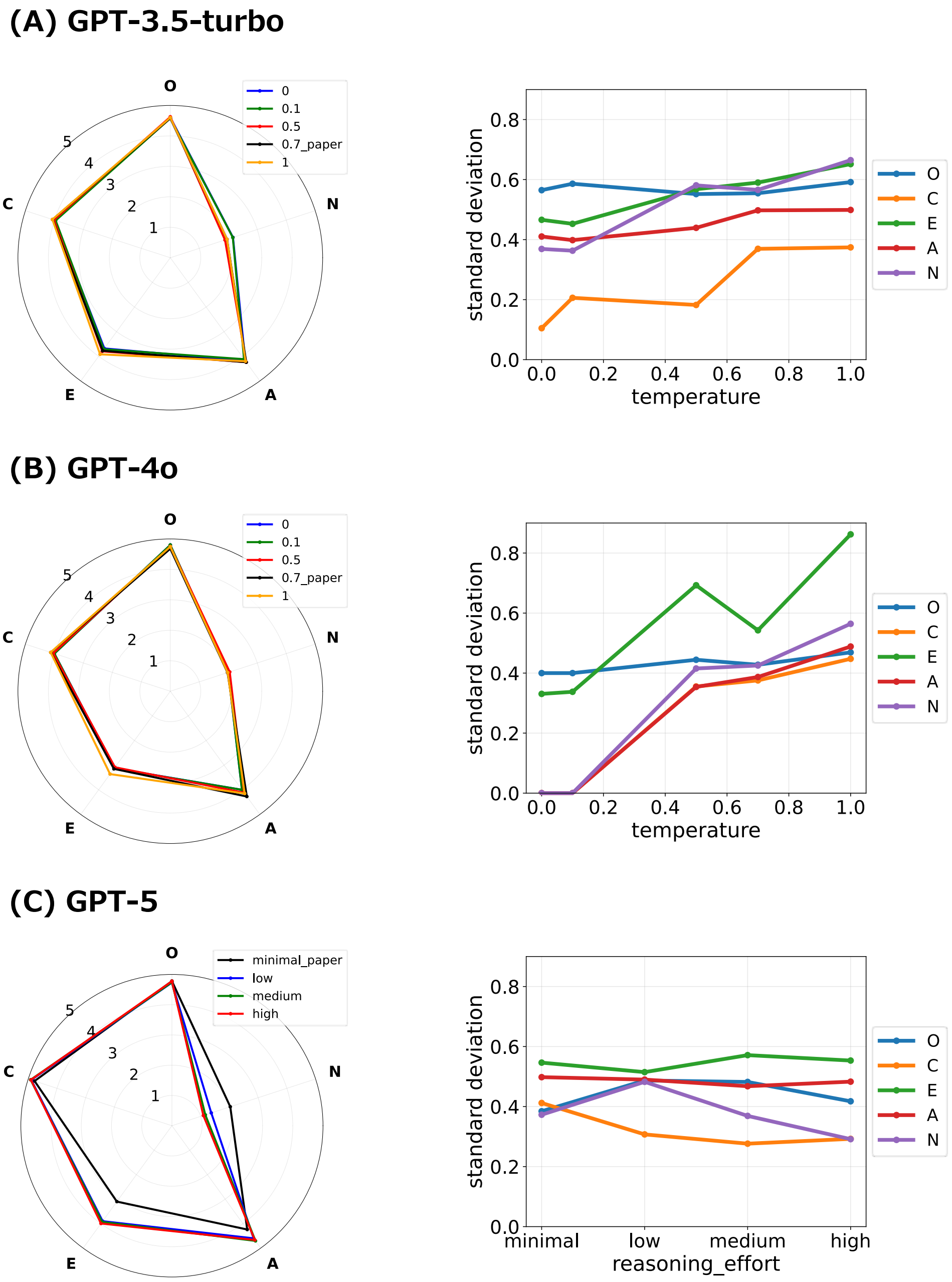

Figure S1: Mean values (radar plots) and standard deviations (line plots) of the Big Five personality traits under different temperature settings (GPT-3.5-turbo and GPT-4o) and reasoning effort levels (GPT-5).

Table S1: Two-way ANOVA results for mean cooperation rate in Experiment 2 across models.

Model	Source	df	F	p
GPT-3.5-turbo	Strategy	4	348.44	$< .001$
	Condition	1	129.40	$< .001$
	Strategy × Condition	4	30.56	$< .001$
	Residual	990	—	—
GPT-4o	Strategy	4	6698.47	$< .001$
	Condition	1	934.80	$< .001$
	Strategy × Condition	4	593.40	$< .001$
	Residual	990	—	—
GPT-5	Strategy	4	1094.60	$< .001$
	Condition	1	115.67	$< .001$
	Strategy × Condition	4	7.63	$< .001$
	Residual	990	—	—

Table S2: Simple effects of condition within each strategy in Experiment 2 (Holm corrected). B_M and P_M denote the mean cooperation rates in the baseline and personality-informed conditions, respectively. Sig. indicates whether the effect is statistically significant after Holm correction.

Model	Strategy	B_M	P_M	t	p (raw)	p (adj)	Sig.
**GPT-3.5-turbo**							
	ALLC	0.972	0.977	-0.47	0.642	0.642	No
	ALLD	0.602	0.774	-12.93	4.12×10^{-28}	2.06×10^{-27}	Yes
	RANDOM	0.745	0.879	-7.48	3.22×10^{-12}	1.29×10^{-11}	Yes
	GRIM	0.964	0.982	-1.46	0.145	0.291	No
	TFT	0.958	0.982	-1.70	0.091	0.274	No
**GPT-4o**							
	ALLC	1.000	1.000	—	—	—	N/A
	ALLD	0.100	0.216	-31.48	2.24×10^{-53}	2.24×10^{-53}	Yes
	RANDOM	0.206	0.743	-25.53	3.76×10^{-62}	7.51×10^{-62}	Yes
	GRIM	1.000	1.000	—	—	—	N/A
	TFT	1.000	1.000	—	—	—	N/A
**GPT-5**							
	ALLC	0.828	0.937	-4.61	1.11×10^{-5}	2.23×10^{-5}	Yes
	ALLD	0.093	0.100	-2.73	0.0075	0.0075	Yes
	RANDOM	0.231	0.410	-6.22	3.16×10^{-9}	1.58×10^{-8}	Yes
	GRIM	0.828	0.952	-4.94	2.86×10^{-6}	8.58×10^{-6}	Yes
	TFT	0.827	0.962	-5.38	4.43×10^{-7}	1.77×10^{-6}	Yes

Table S3: Two-way ANOVA results for personality-informed conditions: GPT-3.5-turbo.

Condition	Source	df	F	p
O1	Strategy	4	277.50	$< .001$
	Condition	1	0.744	.389
	Strategy $\times$ Condition	4	1.010	.401
	Residual	990		
C1	Strategy	4	330.34	$< .001$
	Condition	1	23.48	$< .001$
	Strategy $\times$ Condition	4	3.713	.005
	Residual	990		
E1	Strategy	4	247.04	$< .001$
	Condition	1	122.49	$< .001$
	Strategy $\times$ Condition	4	7.331	$< .001$
	Residual	990		
A1	Strategy	4	98.22	$< .001$
	Condition	1	374.34	$< .001$
	Strategy $\times$ Condition	4	1.194	.312
	Residual	990		
N1	Strategy	4	263.79	$< .001$
	Condition	1	5.248	.022
	Strategy $\times$ Condition	4	1.869	.114
	Residual	990		
O5	Strategy	4	283.38	$< .001$
	Condition	1	1.410	.235
	Strategy $\times$ Condition	4	1.121	.345
	Residual	990		
C5	Strategy	4	230.06	$< .001$
	Condition	1	0.015	.904
	Strategy $\times$ Condition	4	2.605	.035
	Residual	990		
E5	Strategy	4	278.85	$< .001$
	Condition	1	0.336	.562
	Strategy $\times$ Condition	4	0.696	.595
	Residual	990		
A5	Strategy	4	293.28	$< .001$
	Condition	1	5.771	.016
	Strategy $\times$ Condition	4	2.703	.029
	Residual	990		
N5	Strategy	4	280.56	$< .001$
	Condition	1	0.219	.640
	Strategy $\times$ Condition	4	1.036	.387
	Residual	990		

Table S4: Mean cooperation rates and simple effects for conditions with significant interaction (GPT-3.5-turbo). P_M denotes the mean cooperation rate in the personality-informed condition, and C_M denotes the mean cooperation rate under the manipulated condition (scores set to 1 or 5). Sig. indicates whether the effect is statistically significant after Holm correction.

Condition	Strategy	P_M	C_M	t	p (raw)	p (adj)	Sig.
C1	ALLC	0.977	0.965	1.51	0.132	0.397	No
	ALLD	0.774	0.724	3.69	0.00029	0.00143	Yes
	RANDOM	0.879	0.833	2.79	0.0058	0.023	Yes
	GRIM	0.982	0.974	0.99	0.321	0.642	No
	TFT	0.982	0.976	0.84	0.404	0.642	No
E1	ALLC	0.977	0.941	3.17	0.0019	0.0019	Yes
	ALLD	0.774	0.655	7.64	1.07×10^{-12}	5.35×10^{-12}	Yes
	RANDOM	0.879	0.780	5.86	2.08×10^{-8}	8.31×10^{-8}	Yes
	GRIM	0.982	0.924	3.73	0.00028	0.00059	Yes
	TFT	0.982	0.947	3.81	0.00019	0.00059	Yes
C5	ALLC	0.977	0.979	-0.29	0.775	1.000	No
	ALLD	0.774	0.798	-1.78	0.076	0.228	No
	RANDOM	0.879	0.886	-0.45	0.651	1.000	No
	GRIM	0.982	0.966	1.91	0.057	0.228	No
	TFT	0.982	0.962	2.28	0.024	0.119	No
A5	ALLC	0.977	0.977	-5.03×10^{-14}	1.000	1.000	No
	ALLD	0.774	0.795	-1.64	0.102	0.409	No
	RANDOM	0.879	0.914	-2.54	0.012	0.060	No
	GRIM	0.982	0.983	-0.13	0.899	1.000	No
	TFT	0.982	0.979	0.44	0.658	1.000	No

Table S5: Two-way ANOVA results for personality-informed conditions: GPT-4o.

Condition	Source	df	F	p
O1	Strategy	4	2895.64	$< .001$
	Condition	1	7.94	.0049
	Strategy × Condition	4	4.37	.0016
	Residual	990		
C1	Strategy	4	3513.33	$< .001$
	Condition	1	5.51	.0191
	Strategy × Condition	4	6.80	$< .001$
	Residual	990		
E1	Strategy	4	2961.67	$< .001$
	Condition	1	0.06	.802
	Strategy × Condition	4	0.02	.999
	Residual	990		
A1	Strategy	4	1940.06	$< .001$
	Condition	1	52430.97	$< .001$
	Strategy × Condition	4	1940.06	$< .001$
	Residual	990		
N1	Strategy	4	3433.25	$< .001$
	Condition	1	0.21	.644
	Strategy × Condition	4	0.21	.931
	Residual	990		
O5	Strategy	4	3097.49	$< .001$
	Condition	1	0.02	.884
	Strategy × Condition	4	0.01	.999
	Residual	990		
C5	Strategy	4	3293.77	$< .001$
	Condition	1	4.37	.0369
	Strategy × Condition	4	2.64	.0323
	Residual	990		
E5	Strategy	4	3357.03	$< .001$
	Condition	1	12.74	$< .001$
	Strategy × Condition	4	4.78	$< .001$
	Residual	990		
A5	Strategy	4	3751.00	$< .001$
	Condition	1	139.76	$< .001$
	Strategy × Condition	4	53.87	$< .001$
	Residual	990		
N5	Strategy	4	3649.34	$< .001$
	Condition	1	0.23	.633
	Strategy × Condition	4	0.98	.418
	Residual	990		

Table S6: Mean cooperation rates and simple effects for conditions showing significant interaction (GPT-4o). The definitions of P_M, C_M, and Sig. are the same as in Table S4.

Condition	Strategy	P_M	C_M	t	p (raw)	p (adj)	Sig.
O1	ALLC	1.000	1.000	—	—	—	N/A
	ALLD	0.216	0.197	4.68	1.0×10^{-5}	1.4×10^{-5}	Yes
	RANDOM	0.743	0.681	2.18	0.031	0.031	Yes
	GRIM	1.000	1.000	—	—	—	N/A
	TFT	1.000	1.000	—	—	—	N/A
C1	ALLC	1.000	1.000	—	—	—	N/A
	ALLD	0.216	0.279	−9.67	5.4×10^{-18}	1.1×10^{-17}	Yes
	RANDOM	0.743	0.738	0.21	0.834	0.834	No
	GRIM	1.000	1.000	—	—	—	N/A
	TFT	1.000	1.000	—	—	—	N/A
A1	ALLC	1.000	0.000	∞	0.000	0.000	Yes
	ALLD	0.216	0.000	58.62	1.1×10^{-78}	2.2×10^{-78}	Yes
	RANDOM	0.743	0.000	43.98	8.7×10^{-67}	8.7×10^{-67}	Yes
	GRIM	1.000	0.000	∞	0.000	0.000	Yes
	TFT	1.000	0.000	∞	0.000	0.000	Yes
C5	ALLC	1.000	1.000	—	—	—	N/A
	ALLD	0.216	0.205	2.44	0.0158	0.0317	Yes
	RANDOM	0.743	0.698	1.70	0.090	0.090	No
	GRIM	1.000	1.000	—	—	—	N/A
	TFT	1.000	1.000	—	—	—	N/A
E5	ALLC	1.000	1.000	—	—	—	N/A
	ALLD	0.216	0.261	−7.15	2.1×10^{-11}	4.2×10^{-11}	Yes
	RANDOM	0.743	0.789	−1.86	0.064	0.064	No
	GRIM	1.000	1.000	—	—	—	N/A
	TFT	1.000	1.000	—	—	—	N/A
A5	ALLC	1.000	1.000	—	—	—	N/A
	ALLD	0.216	0.334	−14.45	5.0×10^{-30}	1.0×10^{-29}	Yes
	RANDOM	0.743	0.896	−7.14	2.0×10^{-12}	2.0×10^{-11}	Yes
	GRIM	1.000	1.000	—	—	—	N/A
	TFT	1.000	1.000	—	—	—	N/A

Table S7: Two-way ANOVA results for personality-informed conditions: GPT-5.

Condition	Source	df	F	p
O1	Strategy	4	2749.41	$< .001$
	Condition	1	3.06	.0808
	Strategy × Condition	4	6.22	$< .001$
	Residual	990		
C1	Strategy	4	2469.32	$< .001$
	Condition	1	49.02	$< .001$
	Strategy × Condition	4	4.82	$< .001$
	Residual	990		
E1	Strategy	4	2782.18	$< .001$
	Condition	1	65.00	$< .001$
	Strategy × Condition	4	5.52	$< .001$
	Residual	990		
A1	Strategy	4	1357.88	$< .001$
	Condition	1	19522.99	$< .001$
	Strategy × Condition	4	1357.88	$< .001$
	Residual	990		
N1	Strategy	4	2664.89	$< .001$
	Condition	1	34.49	$< .001$
	Strategy × Condition	4	3.58	.0056
	Residual	990		
O5	Strategy	4	2596.24	$< .001$
	Condition	1	13.99	$< .001$
	Strategy × Condition	4	6.70	$< .001$
	Residual	990		
C5	Strategy	4	2564.32	$< .001$
	Condition	1	10.08	.0015
	Strategy × Condition	4	8.17	$< .001$
	Residual	990		
E5	Strategy	4	3048.79	$< .001$
	Condition	1	105.98	$< .001$
	Strategy × Condition	4	26.99	$< .001$
	Residual	990		
A5	Strategy	4	2566.22	$< .001$
	Condition	1	258.37	$< .001$
	Strategy × Condition	4	45.65	$< .001$
	Residual	990		
N5	Strategy	4	3078.39	$< .001$
	Condition	1	20.13	$< .001$
	Strategy × Condition	4	2.39	.0496
	Residual	990		

Table S8: Mean cooperation rates and simple effects for conditions showing significant interaction (GPT-5). The definitions of P_M, C_M, and Sig. are the same as in Table S4.

Condition	Strategy	P_M	C_M	t	p (raw)	p (adj)	Sig.
O1	ALLC	0.937	1.000	-12.98	4.23×10^{-23}	2.12×10^{-22}	Yes
	ALLD	0.100	0.100	0.00	1.000	1.000	No
	RANDOM	0.410	0.367	1.29	0.198	0.397	No
	GRIM	0.952	0.981	-4.52	9.94×10^{-6}	3.97×10^{-5}	Yes
	TFT	0.962	0.974	-1.83	0.0695	0.208	No
C1	ALLC	0.937	0.989	-8.99	4.63×10^{-16}	1.89×10^{-15}	Yes
	ALLD	0.100	0.149	-9.75	3.79×10^{-16}	1.89×10^{-15}	Yes
	RANDOM	0.410	0.513	-3.15	0.00138	0.00345	Yes
	GRIM	0.952	0.978	-3.99	9.55×10^{-5}	2.86×10^{-4}	Yes
	TFT	0.962	0.976	-2.15	0.0324	0.0324	Yes
E1	ALLC	0.937	0.998	-12.07	3.30×10^{-22}	1.65×10^{-21}	Yes
	ALLD	0.100	0.119	-4.82	5.21×10^{-6}	1.04×10^{-5}	Yes
	RANDOM	0.410	0.521	-3.45	6.80×10^{-4}	6.80×10^{-4}	Yes
	GRIM	0.952	0.998	-8.82	1.48×10^{-14}	5.92×10^{-14}	Yes
	TFT	0.962	0.996	-6.46	1.88×10^{-9}	5.63×10^{-9}	Yes
A1	ALLC	0.937	0.000	193.10	$2.176271 \times 10^{-129}$	$6.528812 \times 10^{-129}$	Yes
	ALLD	0.100	0.000	3.584820×10^{16}	0.000000	0.000000	Yes
	RANDOM	0.410	0.000	18.23	2.123510×10^{-33}	2.123510×10^{-33}	Yes
	GRIM	0.952	0.000	189.60	$1.327505 \times 10^{-128}$	$2.655010 \times 10^{-128}$	Yes
	TFT	0.962	0.000	197.20	$2.736859 \times 10^{-130}$	$1.094743 \times 10^{-129}$	Yes
N1	ALLC	0.937	0.993	-10.20	6.68×10^{-19}	3.34×10^{-18}	Yes
	ALLD	0.100	0.100	0.00	1.000	1.000	No
	RANDOM	0.410	0.489	-2.37	0.018	0.038	Yes
	GRIM	0.952	0.990	-6.49	1.01×10^{-9}	4.03×10^{-9}	Yes
	TFT	0.962	0.993	-5.02	8.85×10^{-8}	2.65×10^{-7}	Yes
O5	ALLC	0.937	0.977	-6.21	3.08×10^{-9}	1.54×10^{-8}	Yes
	ALLD	0.100	0.100	0.00	1.000	1.000	No
	RANDOM	0.410	0.498	-2.71	0.0072	0.029	Yes
	GRIM	0.952	0.966	-2.02	0.044	0.133	No
	TFT	0.962	0.949	1.86	0.065	0.133	No
C5	ALLC	0.937	0.991	-9.57	1.83×10^{-17}	9.15×10^{-17}	Yes
	ALLD	0.100	0.100	0.00	1.000	1.000	No
	RANDOM	0.410	0.492	-2.51	0.013	0.039	Yes
	GRIM	0.952	0.951	0.14	0.888	1.000	No
	TFT	0.962	0.937	3.63	3.56×10^{-4}	1.43×10^{-3}	Yes
E5	ALLC	0.937	0.995	-10.89	2.55×10^{-20}	1.28×10^{-19}	Yes
	ALLD	0.100	0.106	-2.51	0.013	0.0356	Yes
	RANDOM	0.410	0.603	-6.39	1.16×10^{-9}	3.48×10^{-9}	Yes
	GRIM	0.952	0.993	-7.27	1.93×10^{-11}	7.73×10^{-11}	Yes
	TFT	0.962	0.991	-5.12	8.62×10^{-7}	1.72×10^{-6}	Yes
A5	ALLC	0.937	1.000	-12.98	4.23×10^{-23}	1.69×10^{-22}	Yes
	ALLD	0.100	0.200	-70.36	2.41×10^{-86}	1.21×10^{-85}	Yes
	RANDOM	0.410	0.687	-8.78	7.91×10^{-16}	2.37×10^{-15}	Yes
	GRIM	0.952	1.000	-9.56	1.00×10^{-16}	2.37×10^{-15}	Yes
	TFT	0.962	1.000	-7.79	6.75×10^{-12}	6.75×10^{-12}	Yes
N5	ALLC	0.937	0.994	-10.54	1.29×10^{-19}	6.43×10^{-19}	Yes
	ALLD	0.100	0.100	0.00	1.000	1.000	No
	RANDOM	0.410	0.426	-0.51	0.616	1.000	No
	GRIM	0.952	0.997	-8.48	6.22×10^{-14}	2.49×10^{-13}	Yes
	TFT	0.962	0.991	-5.12	8.62×10^{-7}	2.59×10^{-6}	Yes
